# *Cryptococcus neoformans* Is Internalized by Receptor-Mediated or ‘Triggered’ Phagocytosis, Dependent on Actin Recruitment

**DOI:** 10.1371/journal.pone.0089250

**Published:** 2014-02-21

**Authors:** Caroline Rezende Guerra, Sergio Henrique Seabra, Wanderley de Souza, Sonia Rozental

**Affiliations:** 1 Laboratório de Biologia Celular de Fungos, Instituto de Biofísica Carlos Chagas Filho, Universidade Federal do Rio de Janeiro, Rio de Janeiro/RJ, Brazil; 2 Laboratório de Tecnologia em Bioquímica e Microscopia, Colegiado de Ciências Biológicas e da Saúde, Centro Universitário Estadual da Zona Oeste, Campo Grande/RJ, Brazil; 3 Instituto Nacional de Metrologia Qualidade e Tecnologia, Duque de Caxias/RJ, Brazil; 4 Instituto Nacional de Biologia Estrutural e Bioimagens, Universidade Federal do Rio de Janeiro, Rio de Janeiro/RJ, Brazil; Instituto de Salud Carlos III, Spain

## Abstract

Cryptococcosis by the encapsulated yeast *Cryptococcus neoformans* affects mostly immunocompromised individuals and is a frequent neurological complication in AIDS patients. Recent studies support the idea that intracellular survival of *Cryptococcus* yeast cells is important for the pathogenesis of cryptococcosis. However, the initial steps of *Cryptococcus* internalization by host cells remain poorly understood. Here, we investigate the mechanism of *Cryptococcus neoformans* phagocytosis by peritoneal macrophages using confocal and electron microscopy techniques, as well as flow cytometry quantification, evaluating the importance of fungal capsule production and of host cell cytoskeletal elements for fungal phagocytosis. Electron microscopy analyses revealed that capsular and acapsular strains of *C. neoformans* are internalized by macrophages via both ‘zipper’ (receptor-mediated) and ‘trigger’ (membrane ruffle-dependent) phagocytosis mechanisms. Actin filaments surrounded phagosomes of capsular and acapsular yeasts, and the actin depolymerizing drugs cytochalasin D and latrunculin B inhibited yeast internalization and actin recruitment to the phagosome area. In contrast, nocodazole and paclitaxel, inhibitors of microtubule dynamics decreased internalization but did not prevent actin recruitment to the site of phagocytosis. Our results show that different uptake mechanisms, dependent on both actin and tubulin dynamics occur during yeast internalization by macrophages, and that capsule production does not affect the mode of *Cryptococcus* uptake by host cells.

## Introduction

The encapsulated yeast *Cryptococcus neoformans* is responsible for human cryptococcosis, affecting mostly immunocompromised individuals. Cryptococcosis occurs via inhalation of fungal cells, with initial establishment of lung infection and subsequent dissemination to other organs, eventually reaching the central nervous system. The disease is the third most frequent neurological complication in AIDS patients and is listed by the CDC as an ‘AIDS-defining condition’. Mortality rates associated with cryptococcosis are still high (55–70%), especially in the developing world [1], [2].

The polysaccharide capsule, composed mostly of glucuronoxylomannan (GXM) and galactoxylomannan (GalXM), is considered an important virulence factor for the medically important species of *Cryptococcus* (*C. neoformans and C. gattii*), since acapsular mutants are less virulent than wild-type strains in murine infections [3]. However, non-encapsulated *C. neoformans* yeasts are still pathogenic for severely immunocompromised hosts, showing the importance of studies with both capsular and acapsular strains [4]. Although capsule production is important for host immune system avoidance by interfering, for example, with cytokine secretion and providing resistance to phagolysosomal enzymes [5], [6], *C. neoformans* also displays capsule-independent mechanisms of escape from immune system cells, such as the expression of anti-phagocytic proteins [7], [8]. However, these mechanisms do not fully prevent phagocytosis through antibody or complement opsonization, or via direct GXM recognition by host cell membrane receptors [9], [10].

In fact, despite classical descriptions of *C. neoformans* as an opportunistic extracellular pathogen, recent studies support the idea that this fungus is a facultative intracellular pathogen displaying relatively sophisticated intracellular survival mechanisms [11], [12]. Internalization of *C. neoformans* by immune system cells and intracellular survival have an important role in the development of cryptococcosis, by facilitating fungal dissemination via a ‘Trojan horse’ phenomenon whereby yeast cells reach different host tissues while ‘hidden’ in the intracellular environment of circulating host cells [13].

Intracellular pathogens are often internalized via a ‘zipper’ mechanism of classical receptor-mediated phagocytosis, where host cell and pathogen surfaces are in close proximity due to specific interactions between surface molecules, required for pathogen engulfment [14]. However, microorganisms also enter host cells by subverting regular endocytic pathways such as clathrin-mediated endocytosis (CME), macropinocytosis and lipid raft/caveolae-dependent endocytosis [15]. For example, microorganisms often ‘trigger’ a macropinocytosis-like phenomenon by secreting proteins that induce host cell membrane ruffling and engulf the pathogen in a large, ‘loose’ vacuole [16]. Both receptor-mediated and triggered phagocytosis require host cell cytoskeletal remodeling [17].

Few studies have examined the role of ‘subverted’ endocytic mechanisms in the phagocytosis of pathogenic fungi, although *Candida albicans* is known to use CME to enter epithelial cells, and *C. neoformans* invades human brain microvascular endothelial cells via lipid raft/caveolae-dependent endocytosis [18]–[20]. *C. neoformans*-macrophage interaction studies to date have focused on intracellular survival and extrusion mechanisms [21], [22]. In contrast, the mechanism of *C. neoformans* internalization by mammalian cells is yet to be described in detail.

In this study, we focused on the initial steps of the interaction between *C. neoformans* and peritoneal macrophages, using confocal fluorescence microscopy, electron microscopy and flow cytometry techniques to address different aspects of the internalization processes, including the participation of the cytoskeleton and the mode of yeast phagocytosis by macrophages. Also, capsular and acapsular mutant yeast strains were used, and we did not opsonize pathogens prior to interaction with host cells, to evaluate the direct effect of capsule formation on *C. neoformans* phagocytosis.

## Materials and Methods

### Fungal Strains

We used the following *Cryptococcus neoformans* strains: the capsular wild type strains H99 used for the genome project (ATCC 208821, serotype A,) and B3501 (serotype D); and the acapsular mutants CAP59 (NE367), derived from H99 [23], and CAP 67 (ATCC 52817), derived from B3501 [24]. All yeast strains were cultured in Sabouraud dextrose agar (Difco, USA) at 35°C.

### Macrophages

Peritoneal macrophages were collected from two-month-old CF1 Swiss mice using Hank’s solution, allowed to adhere for 40 minutes to 13-mm round glass coverslips (for imaging assays) or directly to the wells of 24-well culture plates (for flow cytometry assays), washed with Hank’s solution (to remove unattached cells), and kept in the incubator for 24 h before interaction assays. They were maintained in RPMI 1640 medium with 10% Fetal Bovine Serum (FBS) at 37°C in 5% CO_2_.

### Ethics Statement

The studies were approved by the Ethics Committee of the Carlos Chagas Filho Biophysics Institute (permit number IBCCF 105A). All animals received humane care in compliance with the Principles of Laboratory Animal Care formulated by the National Society for Medical research and the “Guide for the care and use of laboratory animals” prepared by the National Academy of Sciences (Washington, DC, USA).

### Quantification of *C. Neoformans* Attachment and Internalization

Yeast-macrophage interactions were quantified by flow cytometry. Briefly, *C. neoformans* cells were stained with 0.5 mg/ml of Fluorescein isothiocyanate (FITC) (Sigma Chemical Co., Missouri, USA) in phosphate-buffered saline (PBS) for 10 min and incubated with macrophages in a 25∶1 ratio, for 1 h at 4°C. Unattached yeast cells were removed by washing with RPMI medium and internalization was allowed to occur for 2 h at 37°C in 5% CO_2_, and was stopped by the addition of ice-cold PBS.

Macrophages associated with FITC-labelled yeasts were then fixed with 4% paraformaldehyde, gently detached using a cell scraper and analysed for FITC fluorescence in a FACSCalibur flow cytometer (BD Biosciences), the percentage of positive stained events was used to obtain estimates of total yeast-macrophage interactions (combining attachment to macrophages and internalization).

Yeast internalization was quantified according to Chaka *et al* [25] from samples prepared in parallel with those used for the quantification of total yeast-macrophage interactions. Essentially, samples were incubated for 10 min in 0.2 mg/ml of Trypan blue (Merck) in order to quench the FITC fluorescence of attached but non-internalized *C. neoformans*, and then analysed by flow cytometry as described above. The proportion of attached (but not internalized) yeast cells was estimated by subtracting the value for internalized yeasts from the value for total yeast-macrophage interactions. Statistical analyses of internalization and attachment data were performed by two-way ANOVA tests with Dunnett’s multiple comparisons test, using the GraphPad Prism 6.0 software. Differences between samples were considered statistically significant when p<0.05.

### Treatment with Cytoskeleton Inhibitors

To evaluate the importance of host cell cytoskeletal rearrangements during the internalization of *C. neoformans* by macrophages, different inhibitors of actin and microtubule dynamics were added to the medium of 2-hour internalization experiments performed as described above. We used cytochalasin D (0.5 and 1 µM) or latrunculin B (1 and 2 µM) to disrupt the actin cytoskeleton, and nocodazole (5 and 10 µM) or paclitaxel (0.2 and 0.4 µM) to interfere with tubulin dynamics. All compounds were obtained from Sigma Chemical Co. (Missouri, USA). Total yeast-macrophage interactions and yeast internalization were quantified by flow cytometry as described above, and confocal laser scanning microscopy was performed (as described below) for visual inspection of pharmacological effects on cytoskeletal remodeling.

### Yeast Viability Assay

To ensure that cytoskeleton inhibitors were not affecting yeast viability during interaction experiments, *C. neoformans* cells were incubated for 2 h with cytoskeletal inhibitors at the highest concentration used in our studies, or at a 10-fold higher concentration. Afterwards, cells were incubated with 0.4 µM of the FUN®-1 dye (Molecular Probes, Life Technologies Corp.) in HEPES buffer for 20 min at 37°C, and cell viability was assessed by flow cytometry considering the mean fluorescence intensity (FL2 channel). Yeast cells fixed with 70% ethanol were used as a positive control for the loss of cell viability.

### Macrophage Viability Assay

Macrophage viability was assessed after 2 h incubations with cytoskeletal inhibitors at the same concentrations used for yeast viability assays. After drug treatments, macrophages were washed with PBS supplemented with 10 mM glucose and, a mixture of MTS (3-(4,5-dimethylthiazol-2-yl)-5-(3-carboxymethoxyphenyl)-2-(4-sulfophenyl)-2H-tetrazolium, inner salt; MTS) and PMS (phenazine methosulfate) was added to the cells. Macrophage viability was estimated by measuring the absorbance at 490 nm (on a SpectraMax M2e microplate reader, Molecular Devices, US) after 2 h incubation with MTS/PMS at 37°C. As a control for the loss of cell viability, cells were fixed with 4% formaldehyde prior to incubation with MTS/PMS. Three independent experiments were performed.

### Confocal Laser Scanning Microscopy

For analysis by confocal laser scanning microscopy, yeast cells were stained with 0.1 mg/ml calcofluor white (Sigma Chemical Co.) for 20 min prior to interaction assays. Afterwards, cells were fixed in 4% formaldehyde for 30 min and permeabilized with 0.1% saponin in PBS containing 3% Bovine serum albumin (BSA) and 5% fish gelatin. Non-specific labeling sites were blocked with 50 mM ammonium chloride, and then samples were incubated sequentially with a mouse anti-α-tubulin primary antibody (1∶200 dilution) for 1 h and then with anti-mouse secondary antibodies conjugated with Alexa Fluor 488 (1∶400 dilution) for 1 h, with PBS rinses after antibody incubations. Actin filaments were then labeled with phalloidin-Alexa Fluor 546 (1∶40 dilution) for 1 h. Coverslips were mounted onto slides using ProLong Gold antifade reagent with DAPI and examined in a Zeiss 710 LSM confocal microscope. Images created from z-stack series of confocal planes were performed with Zen lite edition 2009 software (Zeiss). All antibodies and fluorescent markers were from Molecular Probes (Life Technologies Corporation).

### Scanning Electron Microscopy (SEM)

For SEM, yeast-macrophage interactions were performed on 13-mm coverslips, in 24-well plates. After interactions, cells were washed and fixed in 2.5% glutaraldehyde and 4% formaldehyde in 0.1 M cacodylate buffer, pH 7.2, for 1 h and post-fixed with 1% OsO_4_ in 0.1 M cacodylate buffer, pH 7.2, and 0.8% potassium ferrocyanide for 1 h. Alternatively, samples containing encapsulated yeasts were fixed with 2.5% glutaraldehde only and post-fixed with 0.2 M sucrose and 2 mM magnesium chloride, for improved capsule preservation. For cytoskeleton observation by SEM, host cell membranes were extracted with 0.5% Triton-X 100 in PHEM buffer, pH 7.2, for 8 min (as described by Svitkina [26]) prior to fixation with 2% glutaraldehyde and sequential 20-min incubations with 0.1% tannic acid and 0.1% uranyl acetate.

After fixation and post-fixation procedures, all samples were dehydrated in an ethanol series (50, 70, 90 and 100%), critical point-dried, mounted onto stubs, sputter coated with gold and observed in a FEI Quanta 250 scanning electron microscope (FEI Company).

SEM samples were used to obtain the percentage of each uptake mechanism by counting the number of uptake events of each type (trigger-like and zipper-like structures) in a total of 200 randomly selected cells from each strain.

### Transmission Electron Microscopy (TEM)

For TEM, cells grown in 60-mm^2^ Petri dishes were fixed, post-fixed and dehydrated as described for SEM, although post-fixation and dehydration were performed in suspension, after gentle cell scraping. Samples were then embedded in Spurr resin (Ted Pella, Inc., USA) and ultrathin sections were stained with uranyl acetate and lead citrate before observation in a JEOL 1200 transmission electron microscope equipped with a CCD camera (Mega View III model, Soft Image System, Germany). Images were processed using the iTEM software (Soft Image System, Germany).

## Results

### Actin and Microtubules Inhibitors Decrease Cryptococcus Internalization by Macrophages

**Figure 1:**
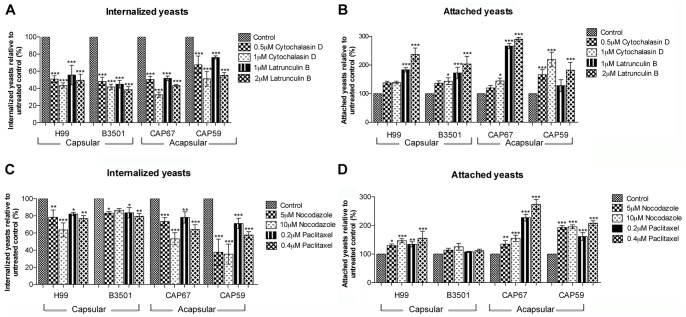
Actin and microtubules inhibitors decrease *C. neoformans* internalization by macrophages. Quantification of the attachment to macrophages and the internalization of *C. neoformans* yeast cells from capsular (H99 and B3501) and acapsular (CAP67 and CAP 59) strains, in the presence of the actin polymerization inhibitors cytochalasin D or latrunculin B (A and B) or the microtubule stabilizers nocodazole or paclitaxel (C and D). Graphs show normalized mean values and standard deviation from three experiments. *p<0.05; **p<0.01; ***p<0.001.

To evaluate the importance of the actin cytoskeleton for the interaction of *C. neoformans* with macrophages, cytochalasin D or latrunculin B were added to the interaction medium. Flow cytometry analysis showed that both compounds decreased the internalization of all four *C. neoformans* strains by macrophages, with corresponding increases in the number of attached (but not internalized) yeast cells (Figure 1, A–B). Cytochalasin D was slightly more effective than latrunculin B at inhibiting yeast internalization, since 1 µM of cytochalasin D inhibited internalization by at least 50%, while treatment with the same concentration of latranculin B resulted in no more than 40% internalization inhibition (Figure 1 A).

Absolute values for internalization were higher for the acapsular strains CAP67 and CAP59 (Figure S1), but normalized data shows that, although CAP59 internalization was somewhat less sensitive to disruption of the actin cytoskeleton, internalization was inhibited to similar extents in all strains, irrespective of capsule production (Figure 1 A).

**Figure 2:**
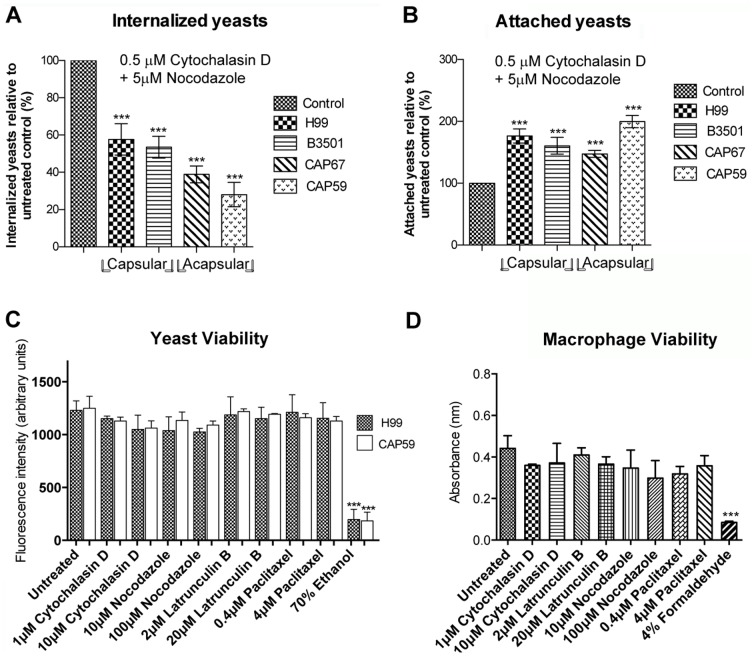
Treatment with both cytochalasin D and nocodazole did not increase the inhibitory effect. Quantification of the internalization (A) and the attachment (B) to macrophages of *C. neoformans* yeast cells from capsular (H99 and B3501) and acapsular (CAP67 and CAP 59) strains, in the absence of cytoskeletal inhibitors or in the presence of cytochalasin D and nocodazole. The metabolic viability of *C. neoformans* strains H99 and CAP59 was measured using the FUN®-1 dye (C) and the metabolic viability of macrophages was measured by MTS/PMS (D) after incubation with cytoskeletal inhibitors for 2 h. Yeast cells fixed with 70% ethanol, and macrophages with 4% formaldehyde, were used as a positive control for the loss of cell viability in each method. Graphs show normalized mean values and standard deviation from three experiments (A–B) and mean and standard deviation from absolute values of fluorescence intensity (C) and absorbance (D).*p<0.05; **p<0.01; ***p<0.001.

Treatment with the microtubule inhibitors nocodazole and paclitaxel resulted in a modest decrease in internalization when compared to that obtained with actin depolymerizing drugs (Figure 1 C). At the concentrations used, paclitaxel was less effective than nocodazole at inhibiting internalization, with 20 to 30% internalization inhibition observed in all strains after paclitaxel treatment (Figure 1 C). In contrast, the negative effect of nocodazole on the internalization of CAP59 yeast cells was stronger than that observed for the other strains (Figure 1 C). As a consequence of this internalization inhibition, the number of yeast cells attached to macrophages was increased (Figure 1 D). Simultaneous disruption of microtubule and actin function by treatment with both cytochalasin D and nocodazole did not increase the inhibitory effect of the individual drugs on yeast internalization (Figure 2, A–B).

Importantly, yeast cells treated with the cytoskeleton inhibitors mentioned above at up to 10-fold higher concentrations than those used in internalization assays remained viable in culture for up to 2 hours (Figure 2C). This figure is representative of yeast cell viability results obtained with all four strains. Similarly, macrophage viability was also unaffected by treatment with cytoskeleton inhibitors for the duration of internalization experiments (Figure 2D). Therefore, the effects of treatment with cytoskeleton inhibitors observed here cannot be explained by drug-induced loss of yeast or macrophage cell viability.

### Actin is Recruited to the Phagosome Area during Cryptococcus Internalization

**Figure 3:**
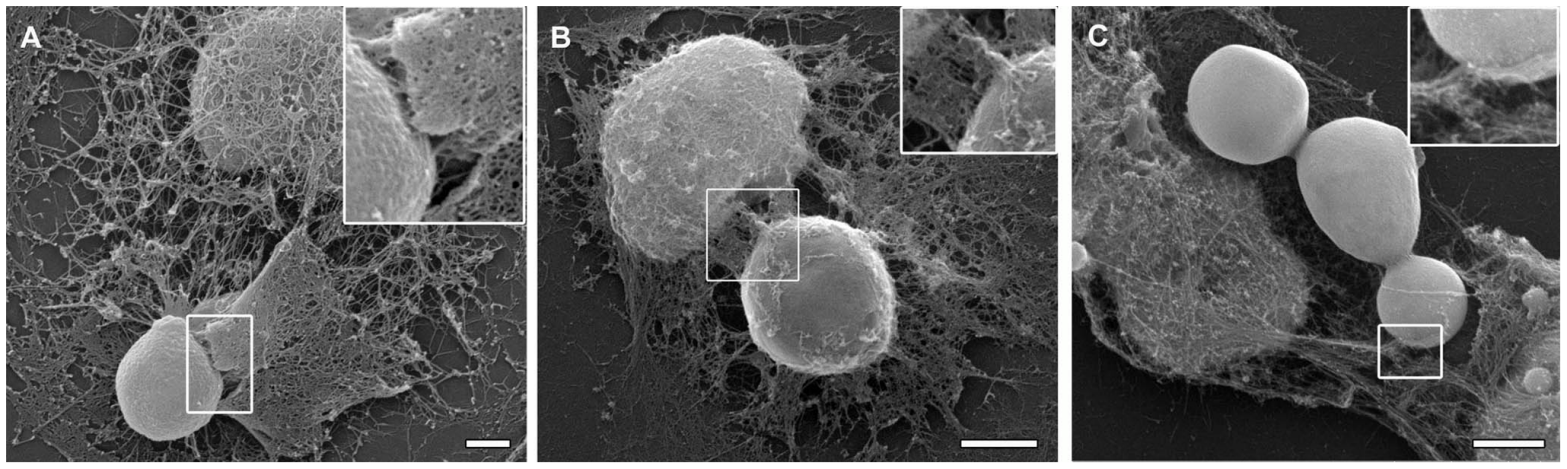
Involvement of the cytoskeleton in the yeast-macrophage interaction. Scanning electron microscopy of membrane extracted macrophages interacting with *C. neoformans* strains H99 (A) and CAP59 (B and C), showing cytoskeletal filaments associated with yeasts in untreated samples (A–B). After 5 µm nocodazole treatment (C) the area surrounding yeast cells appeared mostly devoid of cytoskeletal components but association with yeast still occurred (inset in C). Scale bars, 2 µm.

Initially, we visualized the involvement of the cytoskeleton in the yeast-macrophage interaction by SEM of detergent-extracted cells. In the absence of cytoskeleton inhibitors, filamentous elements surrounded *C. neoformans* capsular and acapsular yeasts associated with macrophages, likely representing cytoskeletal components participating in yeast phagocytosis (Figure 3, A–B). These filaments were disrupted by treatment with cytochalasin D (not shown) independently of the yeast strain used in the interaction. However, nocodazole disrupted filament recruitment to the site of phagocytosis was greater in macrophage interactions with CAP59 cells, where the region surrounding yeast cells appeared largely devoid of cytoskeletal filaments (Figure 3C). This is in agreement with the flow cytometry data showing that the interaction of CAP59 with macrophages was particularly sensitive to nocodazole.

**Figure 4:**
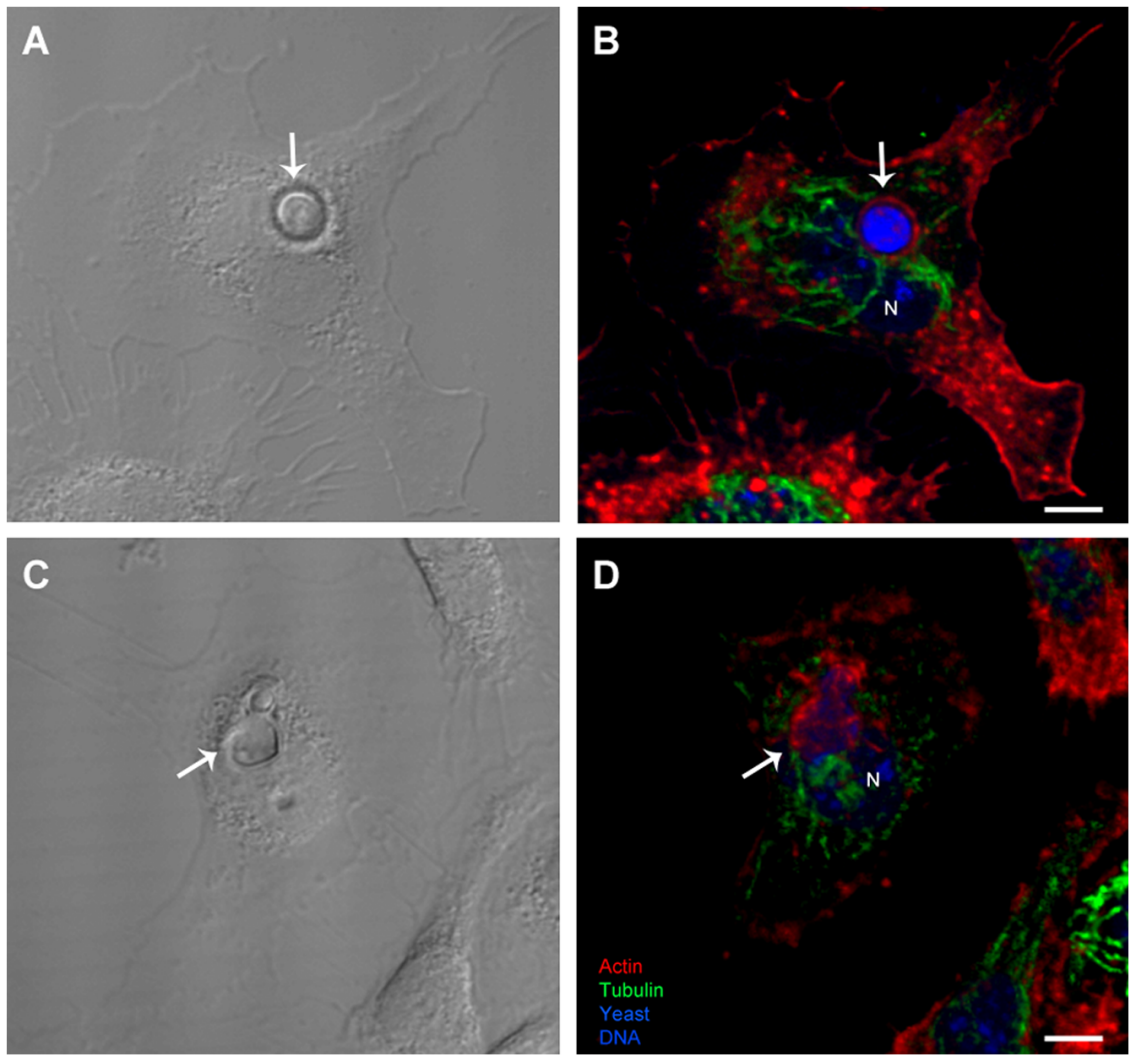
Actin is recruited to the phagosome area during *C. neoformans* internalization. Confocal laser scanning microscopy (z-stack series of confocal planes) of interacting macrophages and *C. neoformans* yeast cells from strains H99 (A and B) and CAP59 (C and D). Internalized yeasts identified by DIC (arrows in A and C) can be visualized in the context of host cell actin (red) and microtubule (green) cytoskeletons (B and D). Host cell DNA is labeled with DAPI (blue, indicated by the letter ‘N’) and yeast is labeled with calcofluor (blue, indicated by arrows). Actin, but not tubulin, is recruited to sites of yeast internalization. Scale bars, 5 µm.

**Figure 5:**
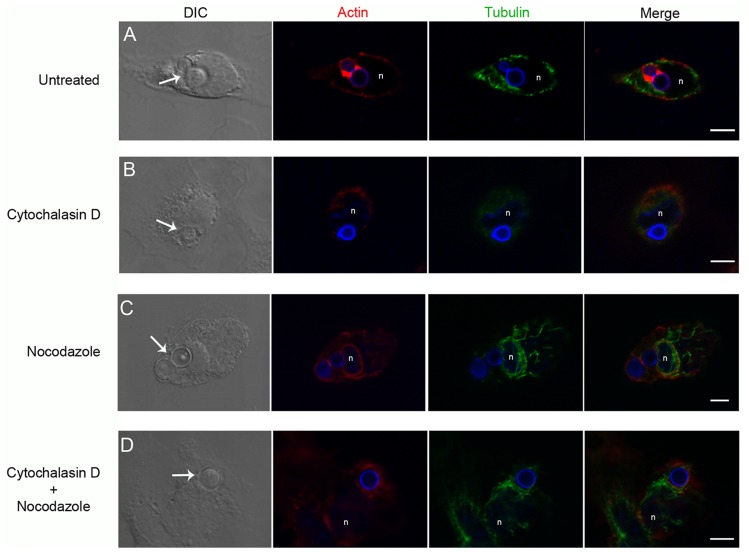
Actin recruitment is inhibited by cytochalasin D. Confocal laser scanning microscopy of *C. neoformans* capsular strain H99 interacting with macrophages (single confocal plane). DIC showing internalized yeasts (arrows); and confocal images showing actin filaments (red), microtubules (green), yeast (blue) and host DNA (blue, indicated by ‘n’). Actin is recruited to the site of phagocytosis in untreated cells (A), and actin recruitment was inhibited by 0.5 µM cytochalasin D (B). In contrast, treatment with 5 µM nocodazole (C) or with a combination of nocodazole and cytochalasin D (D) did not inhibit actin recruitment to the phagosome area. Scale bars, 5 µm.

To determine the nature of the cytoskeletal elements participating in *C. neoformans* internalization by macrophages, actin filaments and microtubules were labeled in samples of yeast-macrophage interactions, and then observed by confocal laser scanning microscopy, for detailed visualization of the labeled cytoskeletal elements. In the absence of inhibitors of cytoskeletal dynamics, actin filaments, but not microtubules, accumulated around internalized yeast cells of both capsular and acapsular strains (Figure 4, B and D). After treatment with cytochalasin D (Figure 5 B) no actin filament accumulation was observed around the site of phagocytosis. Nocodazole treatment did not affect actin recruitment to the phagosome area, although it altered the microtubule distribution throughout the cell (Figure 5 C). Figure 5 D shows the typical host cell cytoskeletal pattern observed when capsular or acapsular strains interacted with macrophages during simultaneous inhibition of actin and tubulin dynamics by cytochalasin D and nocodazole. Interestingly, actin filaments were still recruited to the phagosome area in these samples, despite the presence of cytochalasin D in the medium (Figure 5D).

### Capsular and Acapsular C. Neoformans Strains are Internalized via ‘Zipper’ or ‘Triggered’ Phagocytosis

**Figure 6:**
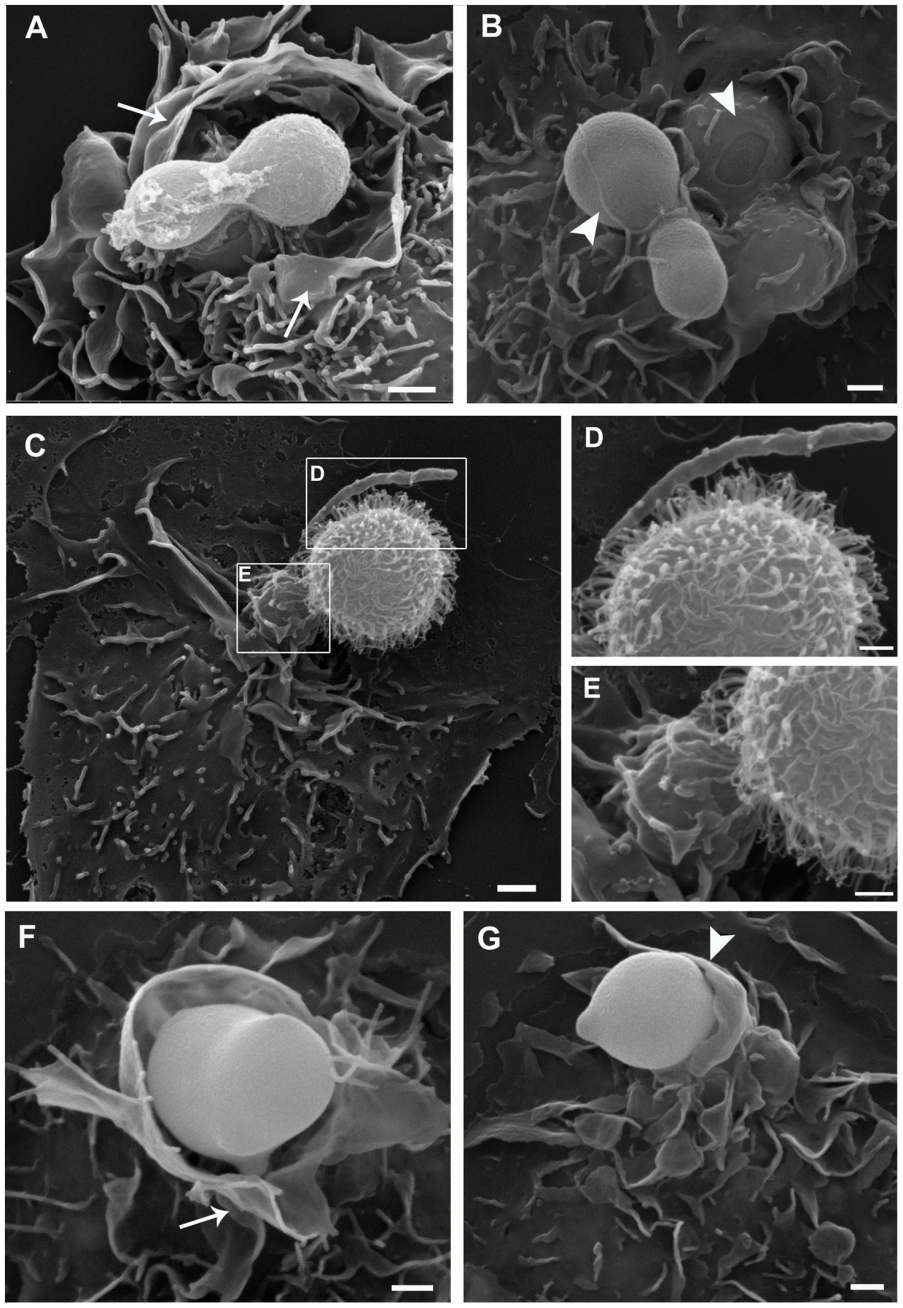
Uptake of *Cryptococcus* strains by trigger-like and zipper-like structures. Scanning electron microscopy of *C. neoformans* capsular strain H99 (A–E) and acapsular strain CAP59 (F–G) interacting with peritoneal macrophages. Improved preservation of macrophage membranes was obtained with routine SEM fixation (A–B; F–G), although post-fixation in the presence of sucrose provided better capsule preservation and allowed visualization of direct interactions between the capsule and host cell membranes, prior to internalization (C–E). Both trigger-like (arrow in A and F) and zipper-like (arrow-head in B and G) uptake structures were observed. Scale bars, 1 µm (A–C; F–G) and 0.5 µm (D–E).

**Figure 7:**
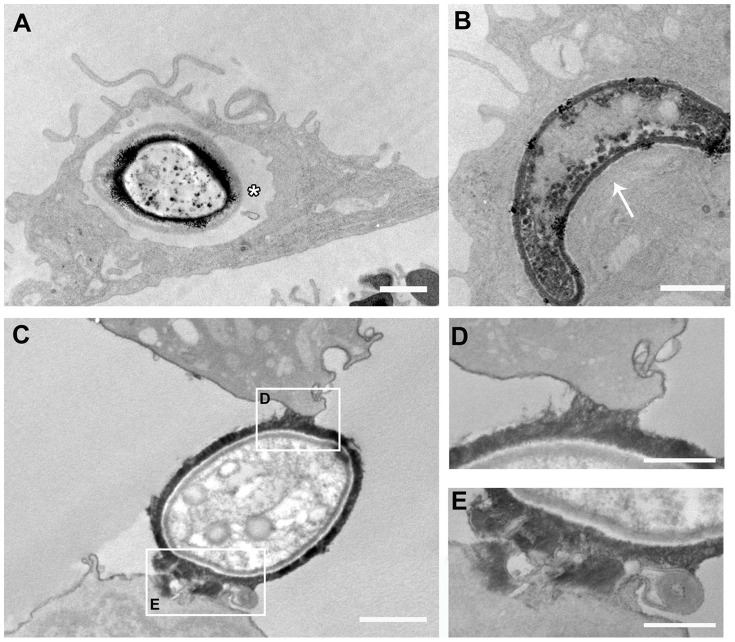
Transmission electron microscopy of *C. neoformans* strain H99 interacting with peritoneal macrophages. Phagosomes of different sizes containing yeast cells were observed in the cytoplasm of macrophages, including ‘loose’ vacuoles typical of ‘triggered’ phagocytosis (asterisk in A), as well as ‘tight’ vacuoles typical of receptor-mediated phagocytosis (arrow in B). We observed direct interactions between the capsule of *C. neoformans* and the macrophage cell membrane (C, and higher magnification in D and E). Scale bars, 1 µm (A–C) and 0.5 µm (D and E).

To evaluate the importance of different phagocytosis mechanisms for the internalization of *C. neoformans* by macrophages, we used SEM and TEM to analyze the morphology of phagocytic structures observed during yeast internalization. Improved structural preservation of macrophage cell membranes, with clear visualization of phagocytic structures, was obtained by using our routine fixation/post-fixation procedure (Figure 6 panels A–B and F–G) as opposed to fixation with glutaraldehyde only and post-fixation with sucrose, which allowed superior yeast capsule preservation (Figure 6 panels C–E). At the site of initial interaction of yeast cells with macrophages, both ruffle-like structures typical of triggered phagocytosis (arrow in Figure 6 A and F), and membrane protrusions tightly involving yeast cells (arrow head in Figure 6 B and G), typical of the zipper-like phenomenon of receptor-mediated phagocytosis, were observed by SEM. Therefore, different uptake mechanisms are involved in the internalization of *C. neoformans* by macrophages and also, quantification of these mechanisms demonstrated a higher percentage of ‘zipper-like’ internalization of both capsular and acapsular yeast (Table 1). Indicating that, even in the absence of opsonization, there is a preference for receptor-mediated phagocytosis irrespective of the presence of a capsule. In samples of capsulated yeasts post-fixed with sucrose for better capsule preservation, the yeast capsule often appeared in direct contact with the host cell membrane, likely due to cell surface molecule interactions (Figure 6 C–E). Also, in TEM images we observed macrophage membrane protrusions, possibly triggered by capsule components, at the site of initial yeast attachment to the macrophage membrane (Figure 7 D and E).

**Table 1:** Quantification of the uptake mechanisms during *C. neoformans*-macrophage interaction.

Strains	Uptake mechanisms (%)*	
	‘Zipper-like’	‘Trigger-like’
H99 (Capsular)	76	24
CAP59 (Acapsular)	64	36

*Percentage obtained by randomly counting 200 cells from each strain.

In agreement with the SEM data on the initial steps of phagocytosis, fully-formed phagosomes examined by TEM were either large and loose, indicating engulfment of yeasts by membrane ruffles (asterisk in Figure 7 A), or tight phagosomes, with membranes in close contact, highly suggestive of zipper-like receptor-mediated entry (arrow in Figure 7 B).

## Discussion

Our work was motivated by the lack of published data on the initial steps of the interaction between non-opsonized *C. neoformans* and host cells. In this study we show the importance of actin recruitment during non-opsonized *C. neoformans* internalization by peritoneal macrophages. Although Cytochalasin D and Latrunculin B inhibit actin polymerization in different ways - the former binding directly to the filament extremity and the latter binding to actin monomers - both drugs caused a significant decrease in yeast internalization, supporting a central role for actin in the internalization of *C. neoformans* by macrophages. Microtubule inhibitors caused a comparatively smaller decrease in internalization, in agreement with the results reported by Chang et al. 2006, showing that the internalization by macrophages of the isolated capsule component GXM was inhibited to a larger extent by actin than by microtubule inhibitors [27]. The effect of cytoskeletal inhibitors on internalization was capsule-independent, because similar inhibition rates were observed for macrophage interactions with encapsulated or acapsular *C. neoformans* strains. Thus, host cells are still capable of recognizing and engulfing encapsulated yeasts in a cytoskeletal-dependent manner, despite the anti-phagocytic properties of capsule components.

Actin plays a crucial role in several processes involving cell membrane dynamics, including endocytosis and receptor-mediated phagocytosis, which trigger intracellular signaling for cytoskeleton and membrane remodeling around the particles or substances being internalized [28]. While actin is important for the formation of the phagocytic cup, microtubule participation in initial pathogen internalization has been reported only for a few bacterial pathogens and it seems to be cell-type and strain specific [29], [30]. Indeed, our immunofluorescence results showing lack of tubulin recruitment around internalized yeasts and limited internalization inhibition by microtubule depolymerizing drugs suggest that microtubules, unlike actin, do not play a central role in the interaction of capsular and acapsular *C. neoformans* with macrophages.

In the field of *C. neoformans* pathogenesis, it is widely believed that the capsule promotes virulence by its anti-phagocytic properties [31]. However, Feldmesser *et al*. [12] have demonstrated that capsular and acapsular *C. neoformans* are internalized efficiently by mouse alveolar macrophages, during experimental intratracheal infections *in vivo*, strongly suggesting that the capsule is not anti-phagocytic. However, it is likely that phagocytosis of capsular and acapsular yeasts in this study occurred via opsonization *in vivo*, a confounding factor in the analysis of the direct role of the capsule in phagocytosis inhibition. In our *in vitro* assays of non-opsonized yeast interactions with macrophages, capsulated yeasts displayed lower internalization rates (represented by lower absolute values for yeast internalization) than genetically related acapsular counterparts (Figure S1). Therefore, our analysis of non-opsonized *C. neoformans* internalization supports the idea that, although the capsule does not completely prevent *C. neoformans* phagocytosis, it does decrease yeast uptake by macrophages. Importantly, our internalization results were performed by flow cytometry analysis of a large (10,000) number of macrophages, and these results were similar when comparing capsular and acapsular strains of two different serotypes, with different capsule structure and composition.

Pathogenic bacteria, fungi and protozoa, as well as viruses, enter host cells by a variety of mechanisms other than receptor-mediated phagocytosis [18], [32]–[34]. Particularly, several intracellular pathogens are known to exploit the phenomenon of macropinocytosis as a nonspecific entry pathway into mammalian host cells [35]. Macropinocytosis is an endocytic mechanism used mostly for fluid-phase uptake of macromolecules, and morphologically characterized by intense membrane ruffling and the formation of large, loose vacuoles. Our SEM and TEM analyses demonstrate that capsular and acapsular *C. neoformans* are internalized by macrophages through at least two different mechanisms. Inside macrophages, *C. neoformans* yeast cells were often found in vacuoles whose membrane was tightly ‘wrapped’ around the yeast, a strong indicator of receptor-mediated phagocytosis by a ‘zipper’ effect of specific interactions between host and yeast surface proteins.

On the other hand, our EM data also shows internalization events with intense macrophage membrane ruffling around yeast cells, as well as the formation of large and loose vacuoles containing pathogens, strongly suggestive of macropinocytosis. In agreement with these observations, Feldmesser et al. [12] demonstrated the ocurrence of cytoplasmic blebs visualized by TEM during the interaction of *C. neoformans* and alveolar macrophages *in vivo.* Thus, it appears that *C. neoformans* enters macrophages by receptor-mediated phagocytosis and, to a lesser extent, by exploiting macropinocytosis as a non-specific phagocytic pathway. Similar results to ours were reported for the interaction of *Aspergillus fumigatus* conidia with macrophages [36], where trigger-like and zipper-like uptake structures were observed by SEM. More experiments are now required to confirm the role of macropinocytosis in *C neoformans* internalization.

Macrophages represent the first line of defense for the immune system and yeast-macrophage interactions are central to cryptococosis pathogenesis. Our results contribute to the understanding of the early steps in this important pathogen-host interaction. We show that non-opsonized *C. neoformans* strains can be internalized by receptor-mediated or non-specific ‘triggered’ phagocytosis, in an actin-dependent and capsule-independent manner. Further studies are ongoing to define if other internalization pathways are also engaged by this pathogenic yeast.

## Supporting Information

Figure S1 **Fluorescence intensity histograms of internalized FITC-stained** ***C. neoformans*** **strains after macrophage interaction.** Cytometry reading after non-opsonized macrophage-*C. neoformans* interaction showed that acapsular strains (CAP59 and CAP67) were greater internalized then capsular strains (H99 and B3501). Percentages indicate positively stained events and show the difference in internalization of genetically related acapsular counterparts: H99 and CAP59 (A); B3501 and CAP67 (B). (MO, macrophage).
(TIF)
