# Supplementary figures and images for: Cryptococcus neoformans Is Internalized by Receptor-Mediated or ‘Triggered’ Phagocytosis, Dependent on Actin Recruitment
Source: PLoS One. 2014 Feb 21;9(2):e89250. doi: 10.1371/journal.pone.0089250 (PMC3931709; doi:10.1371/journal.pone.0089250)

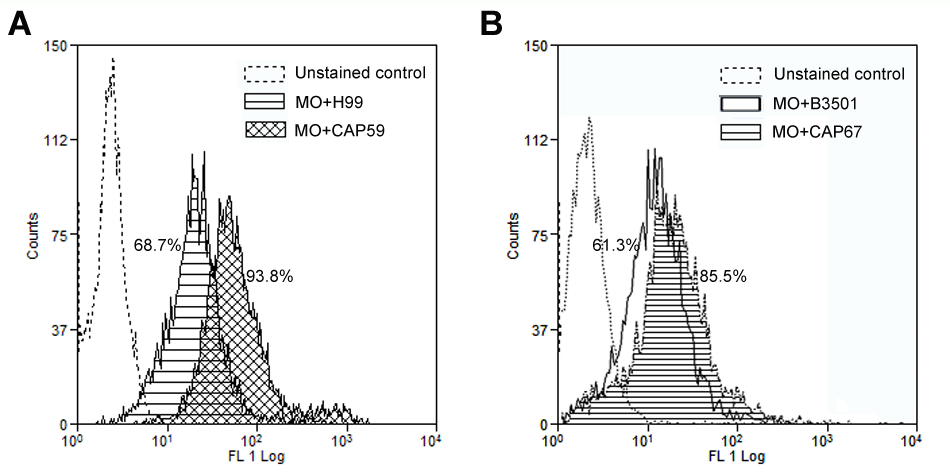

Supplement: Figure S1 — Fluorescence intensity histograms of internalized FITC-stained C. neoformans strains after macrophage interaction. Cytometry reading after non-opsonized macrophage-C. neoformans interaction showed that acapsular strains (CAP59 and CAP67) were greater internalized then capsular strains (H99 and B3501). Percentages indicate positively stained events and show the difference in internalization of genetically related acapsular counterparts: H99 and CAP59 (A); B3501 and CAP67 (B). (MO, macrophage). (TIF) [file pone.0089250.s001.tif]
